# Influence of altered serum and muscle concentrations of BDNF on electrophysiological properties of spinal motoneurons in wild-type and BDNF-knockout rats

**DOI:** 10.1038/s41598-023-31703-8

**Published:** 2023-03-20

**Authors:** Norbert Grzelak, Piotr Krutki, Marcin Bączyk, Dominik Kaczmarek, Włodzimierz Mrówczyński

**Affiliations:** 1 Department of Neurobiology, Poznań University of Physical Education, 27/39 Królowej Jadwigi St., 61-871 Poznań, Poland; 2 Department of Physiology and Biochemistry, Poznań University of Physical Education, Poznań, Poland

**Keywords:** Biochemistry, Biological techniques, Molecular biology, Neuroscience, Physiology, Anatomy, Biomarkers

## Abstract

The purpose of this study was to determine whether altered serum and/or muscle concentrations of brain-derived neurotrophic factor (BDNF) can modify the electrophysiological properties of spinal motoneurons (MNs). This study was conducted in wild-type and *Bdnf* heterozygous knockout rats (HET, SD-BDNF). Rats were divided into four groups: control, knockout, control trained, and knockout trained. The latter two groups underwent moderate-intensity endurance training to increase BDNF levels in serum and/or hindlimb muscles. BDNF and other neurotrophic factors (NFs), including glial cell-derived neurotrophic factor (GDNF), neurotrophin-3 (NT-3), nerve growth factor (NGF), and neurotrophin-4 (NT-4) were assessed in serum and three hindlimb muscles: the tibialis anterior (TA), medial gastrocnemius (MG), and soleus (Sol). The concentrations of tropomyosin kinase receptor B (Trk-B), interleukin-15 (IL-15), and myoglobin (MYO/MB) were also evaluated in these muscles. The electrophysiological properties of lumbar MNs were studied in vivo using whole-cell current-clamp recordings. *Bdnf* knockout rats had reduced levels of all studied NFs in serum but not in hindlimb muscles. Interestingly, decreased serum NF levels did not influence the electrophysiological properties of spinal MNs. Additionally, endurance training did not change the serum concentrations of any of the NFs tested but significantly increased BDNF and GDNF levels in the TA and MG muscles in both trained groups. Furthermore, the excitability of fast MNs was reduced in both groups of trained rats. Thus, changes in muscle (but not serum) concentrations of BDNF and GDNF may be critical factors that modify the excitability of spinal MNs after intense physical activity.

## Introduction

Spinal motoneurons (MNs) control the contractile properties of skeletal muscles and are often targets of adaptive processes resulting from alterations in motor activity. Electrophysiological studies in rats have shown that both decreased (induced by hypokinesis, spinal cord injury, or blocked action potential propagation) and increased (caused by endurance or strength training, muscle overload, or whole-body vibration) levels of locomotive behaviour modify the electrophysiological properties of MNs^1–9^. Adaptive changes in the electrophysiological properties of MNs are believed to be triggered by the activation of specific cellular proteins in response to altered motor demand. The molecular factors responsible for these MN transformations have not been identified, but the involvement of brain-derived neurotrophic factor (BDNF) is often suggested.

BDNF is produced both by brain structures, such as the hippocampus and cerebral cortex, and skeletal muscles, and its levels can change in response to various forms of physical activity^10–16^. MNs express BDNF-responsive tropomyosin kinase receptors (Trks) such as Trk-B^17–20^. Moreover, according to Blum^21^ and Rose et al.^22^, BDNF can rapidly gate Na^+^ channels in the membranes of some types of neurons. Thus, BDNF may be a potential molecular player that induces changes in the electrophysiological properties of spinal MNs.

Nevertheless, it is not clear whether MNs react to BDNF of central or peripheral origin. BDNF levels measured in serum and plasma often increase after endurance exercise in humans^14,23–26^ and rats^27^. Increased levels of post-training BDNF in the bloodstream may be due to its increased expression either in brain structures^14,15^ or skeletal muscles^12,28^. Gardiner^29^ proposed that activity-related changes in muscle BDNF concentrations are responsible for chronic changes in attributes of spinal MNs. Accordingly, BDNF binds to Trk-B receptors and alters the expression of ion channel genes in MNs, controlling their electrophysiological properties. This hypothesis is consistent with the findings of Sagot et al.^30^ and Rind et al.^31^, who reported that BDNF can be retrogradely and trans-synaptically transported from muscles to spinal MNs. In addition, Gonzales and Collins^32^ observed changes in the excitability of fast MNs in rats after exogenous application of BDNF to the MG muscle.

The main goal of the present study was to determine whether altered serum and/or muscle concentrations of BDNF are related to changes in the electrophysiological properties of lumbar MNs in rats. For this purpose, four separate groups of animals were studied: (1) control rats (*Bdnf*+/+), (2) *Bdnf* knockout rats (HET, SD-BDNF) (*Bdnf*+/−), (3) trained control rats (5 weeks of endurance running exercises performed to induce increases in serum and/or muscle BDNF levels [*Bdnf*+/+T]), and (4) trained knockout rats that were subjected to the same training regimen (*Bdnf*+/−T). In each group, the passive, threshold, and rhythmic firing properties of MNs were investigated. Subsequently, the levels of myokines indicating the degree of muscle contractile activity [interleukin-15 (IL-15) and myoglobin (MYO/MB)]; BDNF and its receptor, Trk-B; and the most widespread NFs [glial cell-derived neurotrophic factor (GDNF), neurotrophin-3 (NT-3), nerve growth factor (NGF), and neurotrophin-4 (NT-4)] were measured in serum (NFs only) and three rat hindlimb muscles acting as ankle flexors or extensors: the tibialis anterior (TA), medial gastrocnemius (MG), and soleus (Sol).

## Results

### Body and muscle weights

Significant differences were found between the mean body weights of *Bdnf*+/+ (524.7 ± 16.7 g) and *Bdnf*+/− (681.4 ± 74.4 g) rats measured on the day of the electrophysiological experiment (F_1,33_ = 56.84, p < 0.0001, post hoc test p = 0.0002). Animals in the *Bdnf*+/+ T and *Bdnf*+/− T groups weighed less than those in the *Bdnf*+/+ and *Bdnf*+/− groups by an average of 39.9 g (F_1,33_ = 14.76, p = 0.0005, post hoc test p = 0.33) and 92.8 g (F_1,33_ = 14.76, p = 0.0005, post hoc test p = 0.0051), respectively.

There were also significant differences in the average weights of the TA muscles of the *Bdnf*+/+ and *Bdnf*+/− animals (0.9 ± 0.06 g vs. 1.1 ± 0.2 g, F_1,33_ = 14.90, p = 0.0005, post hoc test p = 0.0086), but not in those of the MG and Sol muscles. Endurance training did not cause considerable changes in the weights of any of the muscles tested in the *Bdnf*+/+ T or *Bdnf*+/− T animals (p > 0.05). However, endurance exercise did significantly increase the muscle-to-body weight ratio of the TA muscle from 0.0017 ± 0.0002 in the *Bdnf*+/+ group to 0.002 ± 0.0002 in the *Bdnf*+/+ T group (F_1,33_ = 24.34, p < 0.0001, post hoc test p = 0.0031), and from 0.0016 ± 0.0002 in the *Bdnf*+/− group to 0.0019 ± 0.0001 in the *Bdnf*+/− T group (F_1,33_ = 24.34, p < 0.0001, post hoc test p = 0.0154). Similar results were observed for the MG muscle, from 0.0019 ± 0.0002 in the *Bdnf*+/− group to 0.0023 ± 0.0003 in the *Bdnf*+/− T group (F_1,33_ = 16.24, p = 0.0003, post hoc test p = 0.00148). However, no changes in this parameter resulting from endurance exercise were found for the Sol muscle (p > 0.05).

### Concentrations of NFs in serum

**Figure 1:**
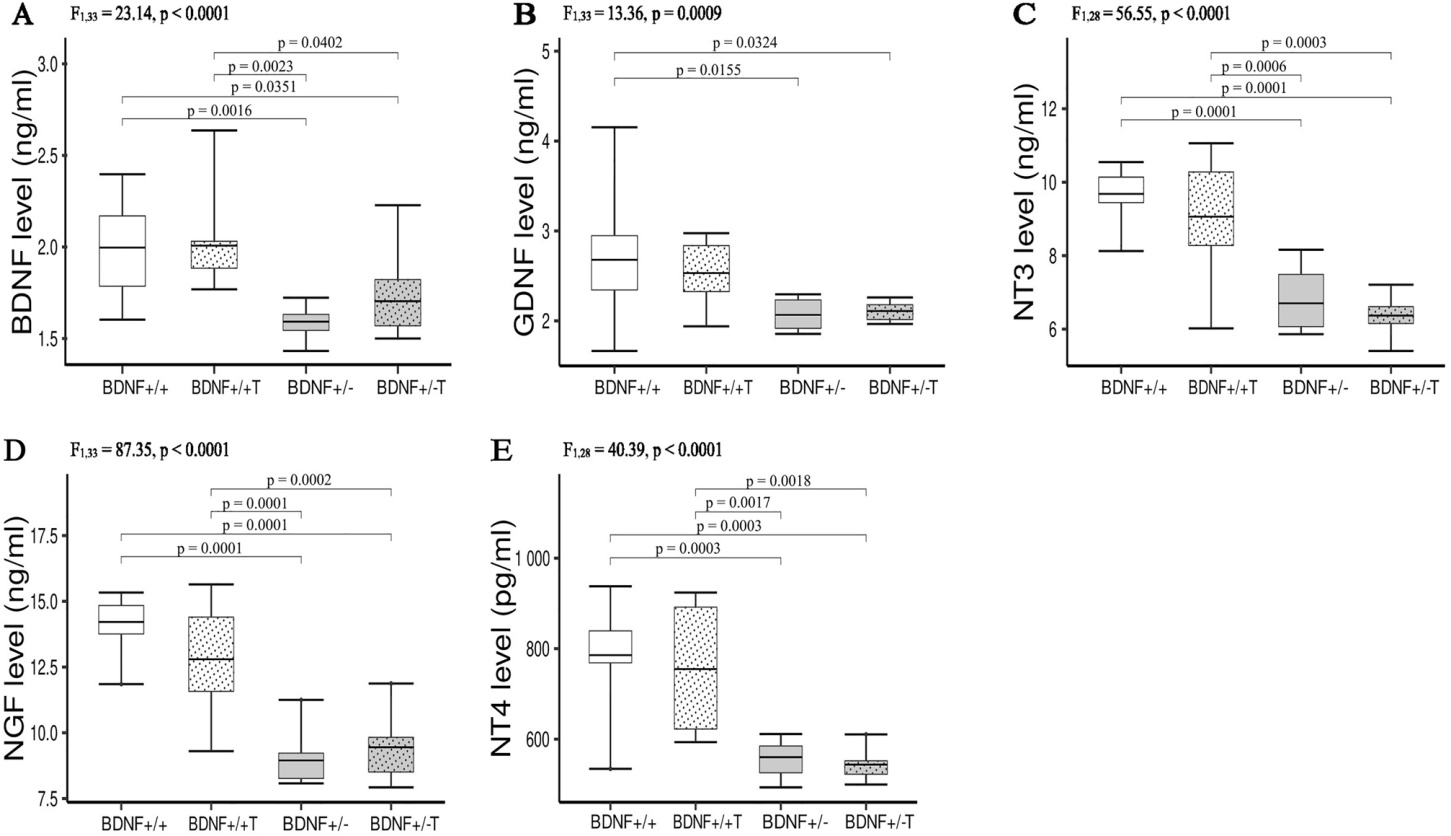
The concentrations of BDNF (**A**), GDNF (**B**), NT3 (**C**), NGF (**D**) and NT4 (**E**) in the serum of *Bdnf*+/+, *Bdnf*+/+ T, *Bdnf*+/− , and *Bdnf*+/− T rats. The bars indicate the mean values, box-plots ± 25% of the dataset, and whiskers ± SD. Differences between the *Bdnf*+/+ and *Bdnf*+/− animals, as well as between the *Bdnf*+/+ T and *Bdnf*+/− T groups are indicated above the plots (two-way ANOVA with genotype and training as fixed factors with Tukey’s HSD post-hoc tests).

The serum of genetically modified rats (*Bdnf* + / −) had significantly lower concentrations of BDNF (by 20%), GDNF (by 22%), NT-3 (by 31%), NGF (by 37%), and NT-4 (by 29%) than the serum of *Bdnf*+/+ rats (Fig. 1A–E). Significantly lower concentrations of BDNF (by 15%), NT-3 (by 30%), NGF (by 26%), and NT-4 (by 28%) were also found in the trained group of genetically modified animals (*Bdnf*+/− T) compared to trained animals in the *Bdnf*+/+ T group (Fig. 1A–E). Surprisingly, 5 weeks of endurance training on a treadmill did not cause significant changes in the serum levels of any of the NFs tested in rats from both trained groups (Fig. 1A–E).

### Concentrations of NFs in hindlimb muscles

**Figure 2:**
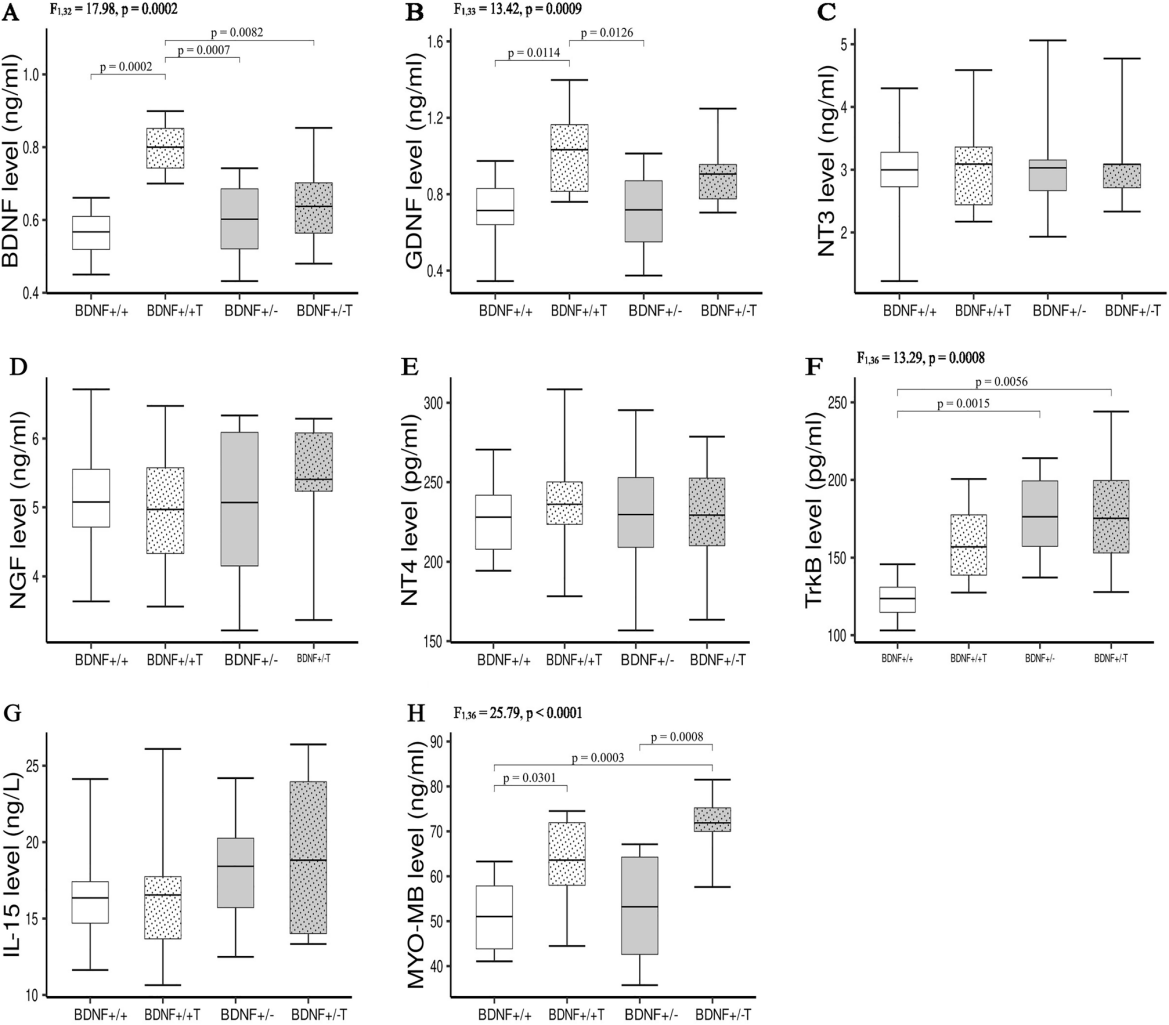
The concentrations of BDNF (**A**), GDNF (**B**), NT3 (**C**), NGF (**D**), NT4 (**E**), TrkB (**F**), IL-15 (**G**), MYO-MB (**H**) in tibialis anterior muscles of *Bdnf*+/+, *Bdnf*+/+ T, *Bdnf*+/−, and *Bdnf*+/− T rats. The bars indicate the mean values, box-plots ± 25% of the dataset, and whiskers ± SD. Differences between the *Bdnf*+/+ and *Bdnf*+/− animals, as well as between the *Bdnf*+/+ T and *Bdnf*+/− T groups are indicated above the plots (two-way ANOVA with genotype and training as fixed factors with Tukey’s HSD post-hoc tests).

**Figure 3:**
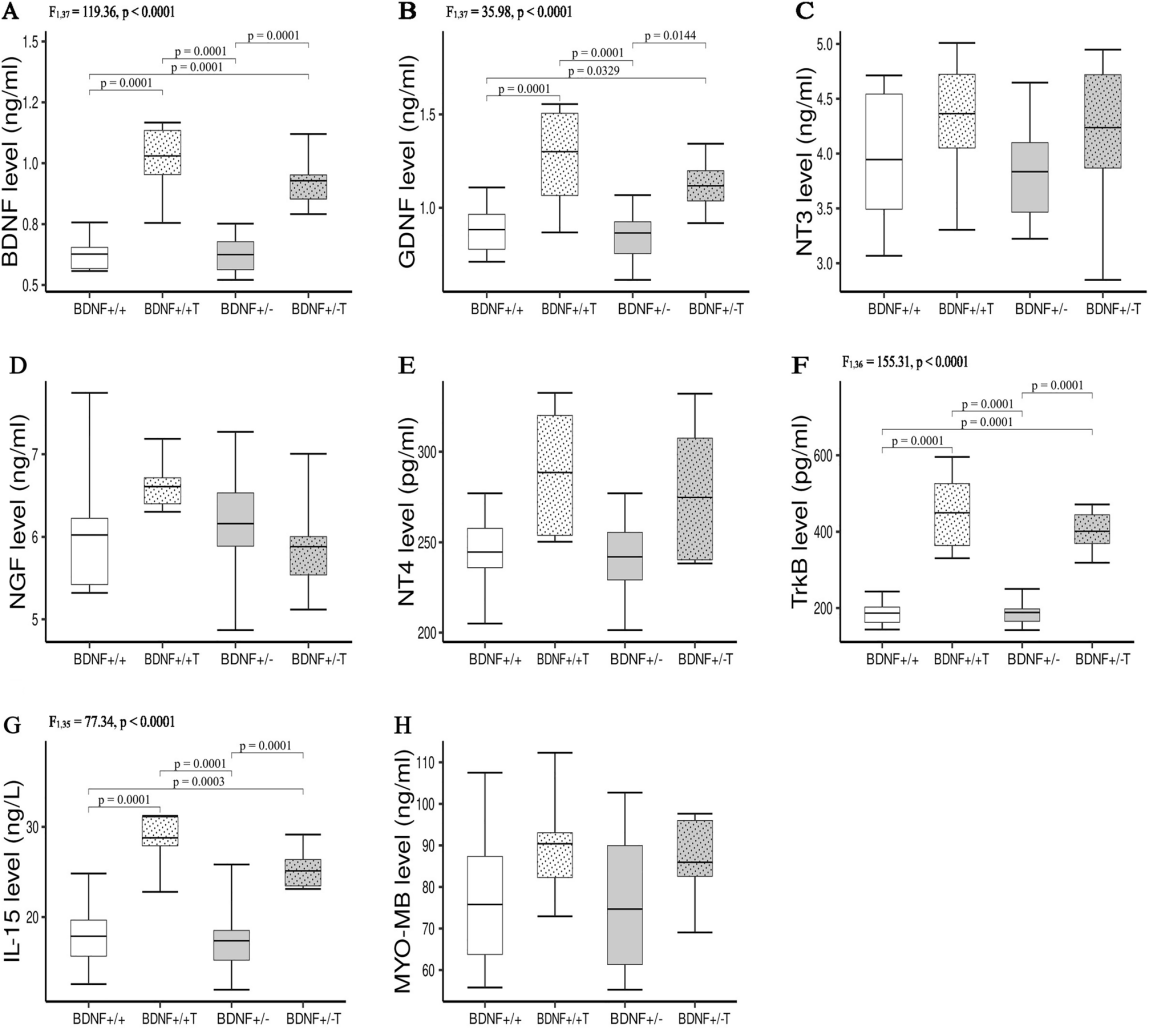
The concentrations of BDNF (**A**), GDNF (**B**), NT3 (**C**), NGF (**D**), NT4 (**E**), TrkB (**F**), IL-15 (**G**), MYO-MB (**H**) in medial gastrocnemius muscles of *Bdnf*+/+, *Bdnf*+/+ T, *Bdnf*+/−, and *Bdnf*+/− T rats. The bars indicate the mean values, box-plots ± 25% of the dataset, and whiskers ± SD. Differences between the *Bdnf*+/+ and *Bdnf*+/− animals, as well as between the *Bdnf*+/+ T and *Bdnf*+/− T groups are indicated above the plots (two-way ANOVA with genotype and training as fixed factors with Tukey’s HSD post-hoc tests).

**Figure 4:**
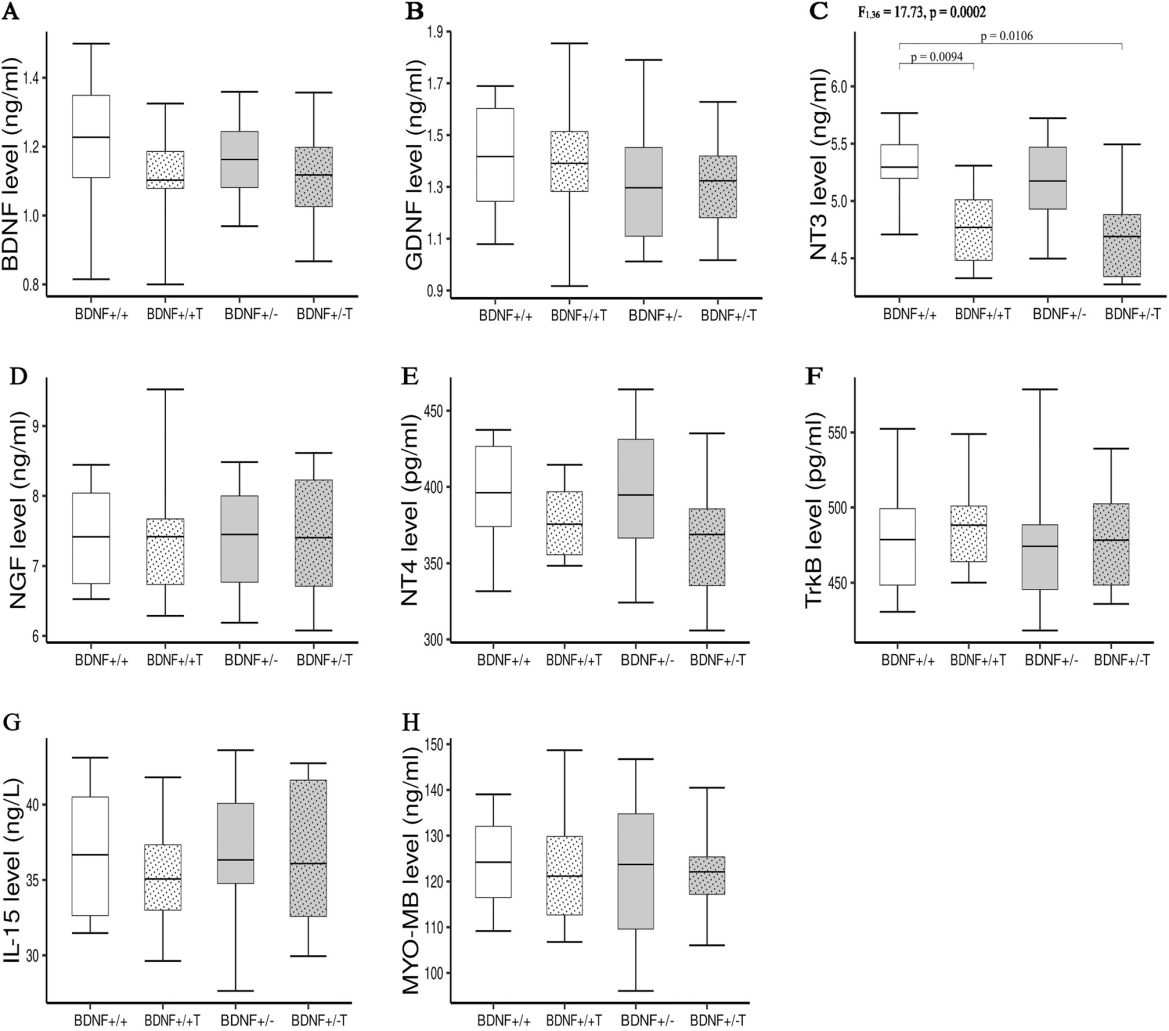
The concentrations of BDNF (**A**), GDNF (**B**), NT3 (**C**), NGF (**D**), NT4 (**E**), TrkB (**F**), IL-15 (**G**), MYO-MB (**H**) in soleus muscles of *Bdnf*+/+, *Bdnf*+/+ T, *Bdnf*+/−, and *Bdnf*+/− T rats. The bars indicate the mean values, box-plots ± 25% of the dataset, and whiskers ± SD. Differences between the *Bdnf*+/+ and *Bdnf*+/− animals, as well as between the *Bdnf*+/+ T and *Bdnf*+/− T groups are indicated above the plots (two-way ANOVA with genotype and training as fixed factors with Tukey’s HSD post-hoc tests).

In contrast to the results observed in serum, the levels of all tested NFs in the TA, MG, and Sol muscles (BDNF, GDNF, NT-3, NGF, and NT-4) of *Bdnf*+/+ and *Bdnf*+/− rats, as well as *Bdnf*+/− T and *Bdnf*+/− T rats, were not statistically different (Figs. 2, 3 and 4A–E). In addition, there were no differences in IL-15 or MYO/MB content across the three tested muscles of *Bdnf*+/+ and *Bdnf*+/− rats as well as *Bdnf*+/− and *Bdnf*+/− T rats (Figs. 2, 3 and 4G,H). A statistically significant difference in the concentration of Trk-B was found between the TA muscles of *Bdnf*+/+ and *Bdnf*+/− rats (Fig. 2F), but not between the MG or Sol muscles (Figs. 3 and 4F).

On the other hand, five weeks of endurance training significantly increased the concentrations of BDNF, GDNF, Trk-B, and IL-15 in the MG muscles of rats in the *Bdnf*+/+ T group compared to the *Bdnf*+/+ group (Fig. 3A,B,F,G), as well as in the MG muscles of rats in the *Bdnf*+/− T group compared to the *Bdnf*+/− group (Fig. 3A,B,F,G). The levels of the other NFs (NT-3, NT-4, and NGF) and MYO/MB did not change in this muscle after endurance training (Fig. 3C–E,H). In the TA muscle, endurance training induced a significant increase in the concentrations of BDNG, GDNF, and MYO/MB in the *Bdnf*+/+ T group compared to the *Bdnf*+/+ group (Fig. 2A,B,H), and increased the level of MYO/MB in the *Bdnf*+/− T group compared to the *Bdnf*+/− group (Fig. 2H). Levels of the remaining NFs (NT-3, NGF, and NT-4), Trk-B, and IL-15 were not changed in the TA muscles of animals in the trained groups (Fig. 2C–G). Interestingly, no significant post-training changes were found in the Sol muscles of the *Bdnf*+/+ T and *Bdnf*+/− T groups compared to their respective controls (Fig. 4A,B,D–H), with the exception of a decrease in NT-3 in the *Bdnf*+/+ T group compared to the *Bdnf*+/+ group (Fig. 4C).

### Electrophysiological properties of fast and slow MNs

Electrophysiological recordings from a total of 174 lumbar MNs were analysed: 42 in the *Bdnf*+/+ group (28 classified as fast and 14 as slow), 47 in the *Bdnf*+/− group (33 classified as fast and 14 as slow), 41 in the *Bdnf*+/+ T group (26 classified as fast and 15 as slow), and 44 in the *Bdnf*+/− T group (29 classified as fast and 15 as slow). There were no statistically significant differences in the electrophysiological properties of any fast- or slow-type MN (p > 0.05, Table 1) between rats of different genotypes (*Bdnf*+/+ and *Bdnf*+/−). However, 5 weeks of endurance training on a treadmill induced significant decreases in the R_IN_ values of fast MNs from *Bdnf*+/+ T and *Bdnf*+/− T animals compared to their respective controls (*Bdnf*+/+ and *Bdnf*+/− groups) (F_1,112_ = 30.03, p < 0.0001, post hoc test p = 0.0081; F_1,112_ = 30.03, p < 0.0001, post hoc test p = 0.0066, respectively; Table 1). In addition, there were trends toward increased rheobase values (F_1,112_ = 8.27, p = 0.0048, post-hoc test p = 0.1367; F_1,112_ = 8.27, p = 0.0048, post-hoc test p = 0.2402), the minimum steady-state firing (SSF) current (F_1,60_ = 2.78, p = 0.1009, post-hoc test p = 0.8563; F_1,60_ = 2.78, p = 0.1009, post-hoc test p = 0.4003), and the maximum SSF current (F_1,60_ = 4.72, p = 0.0338, post-hoc test p = 0.7692; F_1,60_ = 4.72, p = 0.0338, post-hoc test p = 0.1565).

**Table 1:** The mean values (± SD) of passive, threshold, and rhythmic firing properties of fast and slow MNs from *Bdnf*+/+, *Bdnf*+/+ T, *Bdnf*+/−, and *Bdnf*+/− T rats.

	Passive and threshold properties of MNs									Rhythmic firing properties of MNs				
	RMP (mV)	AP_amp_ (mV)	AP_half-width_ (ms)	AHP_peak time_ (ms)	AHP_amp_ (mV)	AHP_hdt_ (ms)	R_IN_ (MΩ)	Rheo (nA)	VT (mV)	Min SSF current (nA)	Min SSS frequency (Hz)	Max SSF current (nA)	Max SSF frequency (Hz)	f–I slope
Fast MNs														
* Bdnf*+/+ (n = 28)	−64.7 ± 8.8	73.7 ± 13.1	0.53 ± 0.1	8.0 ± 2.0	3.5 ± 0.8	12.1 ± 1.8	2.3 ± 0.6	8.2 ± 3.4	−46.6 ± 9.9	12.6 ± 4.8	30.6 ± 8.9	26.4 ± 8.0	81.3 ± 33.8	3.8 ± 1.4
* Bdnf*+/+ T (n = 26)	−63.4 ± 8.0	72.1 ± 12.8	0.53 ± 0.1	8.0 ± 1.7	3.3 ± 0.6	12.2 ± 1.3	1.7 ± 0.5 ^##^	10.6 ± 4.5	−45.5 ± 7.8	14.1 ± 5.1	32.8 ± 10.1	29.4 ± 9.5	80.4 ± 21.5	3.1 ± 0.9
* Bdnf*+/− (n = 33)	−68.7 ± 6.1	74.5 ± 11.8	0.58 ± 0.1	7.9 ± 2.1	3.4 ± 0.7	12.0 ± 1.5	2.4 ± 0.8	8.4 ± 3.6	−48.8 ± 9.2	12.3 ± 4.2	31.0 ± 7.2	25.7 ± 9.2	82.3 ± 23.2	3.9 ± 1.3
*Bdnf*+/− T (n = 29)	−64.5 ± 8.1	73.8 ± 9.4	0.55 ± 0.1	8.2 ± 1.5	3.3 ± 0.6	12.4 ± 1.3	1.7 ± 0.5 ^###^	10.3 ± 4.4	−47.2 ± 6.4	15.1 ± 6.3	30.9 ± 10.6	32.2 ± 8.2	86.5 ± 26.5	3.2 ± 1.1
Slow MNs														
* Bdnf*+/+ (n = 14)	−62.1 ± 6.4	71.5 ± 11.4	0.57 ± 0.1	8.8 ± 2.7	6.2 ± 1.8	21.3 ± 1.0	3.3 ± 0.5	2.6 ± 1.0	−53.1 ± 6.5	3.9 ± 1.8	24.2 ± 8.3	13.4 ± 5.0	68.9 ± 31.9	5.0 ± 2.0
* Bdnf*+/+ T (n = 15)	−59.0 ± 9.3	64.4 ± 6.8	0.49 ± 0.04	8.2 ± 2.2	5.6 ± 0.8	20.8 ± 0.7	3.3 ± 0.4	2.0 ± 0.9	−52.3 ± 8.6	4.3 ± 1.9	30.0 ± 11.7	11.9 ± 4.5	61.8 ± 19.6	4.8 ± 2.3
* Bdnf*+/− (n = 14)	−61.8 ± 6.8	68.6 ± 10.9	0.58 ± 0.1	8.5 ± 3.3	5.1 ± 1.4	21.0 ± 0.7	3.2 ± 0.5	2.6 ± 1.0	−51.9 ± 7.7	5.4 ± 2.7	26.0 ± 10.4	15.9 ± 4.7	70.6 ± 15.8	5.0 ± 1.8
* Bdnf*+/− T (n = 15)	−62.5 ± 5.9	67.7 ± 12.4	0.56 ± 0.1	6.9 ± 1.3	5.3 ± 0.9	20.9 ± 0.8	3.4 ± 0.6	2.4 ± 1.0	−54.2 ± 6.0	4.1 ± 1.6	23.8 ± 4.5	12.0 ± 4.2	55.7 ± 10.4	4.5 ± 1.7

Differences between *Bdnf*+/− and *Bdnf*+/− T rats are statistically significant at ^##^p < 0.01; ^###^p < 0.001 (two-way ANOVA with genotype and training as fixed factors and Tukey’s HSD post-hoc test).

*n* number of MNs, *RMP* resting membrane potential, *AP*_*amp*_ action potential amplitude, *AP*_*half-width*_ action potential duration measured at the level of half-amplitude, *AHP*_*peak time*_ time to afterhyperpolarization (AHP) peak, *AHP*_*amp*_ AHP amplitude, *AHP*_*hdt*_ AHP half-decay time, *R*_*IN*_ input resistance, *Rheo* rheobase, *VT* voltage threshold, *min steady-state firing (SSF) current* minimum current evoking SSF, *min SSF frequency* minimum frequency of the SSF, *max SSF current* maximum current evoking SSF, *max SSF frequency* maximum frequency of SSF, *f–I slope* slope of the f–I relationship.

## Discussion

This study revealed several unexpected findings. We hypothesized that reduced serum BDNF levels would be the only factor to modify the properties of spinal MNs in knockout rats. However, reduced serum levels of all NFs tested were detected in rats with only one functional *Bdnf* allele (their levels were unchanged in muscles); therefore, a combined effect of all serum-reduced NFs on MNs was observed. However, lower serum NF levels had no effects on the passive, threshold, or rhythmic firing properties of fast or slow spinal MNs in rats.

Numerous meta-analyses have shown that BDNF levels in serum and plasma can significantly increase after different forms of endurance activity^33–35^. However, moderate-intensity endurance training on a treadmill, which was used in our study, did not raise the levels of any NFs tested in serum, but significantly increased the concentrations of BDNF and GDNF in fast hindlimb muscles (TA and MG) of both wild-type and knockout rats. A major effect of the observed post-training increases in muscle BDNF and GDNF was a decrease in the excitability of fast MNs, which were already less excitable than the slow MNs^36^.

*Bdnf*+/− rats have been previously used in numerous studies of psychiatric and neurodegenerative diseases associated with significant deficits in peripheral and central BDNF protein^37–39^. Therefore, these animals were selected as a model in our study to investigate changes in the electrophysiological properties of spinal MNs exposed to low concentrations of BDNF. Previous work has demonstrated that BDNF concentrations can be reduced by 73% and 50% in the serum and frontal cortex, respectively, of these animals compared to control rats^37,38^. Significantly lower (20%) serum BDNF levels in *Bdnf* heterozygotes were also noted in our study. This finding coincided with lower levels of other NFs (GDNF, NT-3, NGF, and NT-4) in knockout compared to control rats. This result indicates that the absence of one *Bdnf* allele leads to reductions in the serum concentrations of several major NFs. Due to the existence of separate genes encoding different NFs^40^, we conclude that normal expression levels of *Bdnf* are necessary to maintain proper serum levels of the other NFs.

Our results also indicate that the decreased serum levels of all NFs in *Bdnf*+/− rats were not due to their reduced expression in muscles. The levels of BDNF, GDNF, NT-3, NGF, and NT-4 (as well as those of IL-15 and MYO/MB) in the TA, MG, and Sol muscles were comparable between *Bdnf*+/+ and *Bdnf*+/− rats (Figs. 1, 2, 3 and 4). Since the membrane parameters and firing properties of fast and slow MNs in *Bdnf*+/− rats were not different from those in *Bdnf*+/+ rats (Table 1), the lower serum concentrations of BDNF, GDNF, NT-3, NGF, and NT-4, with their unchanged levels in hindlimb muscles, likely do not modify the electrophysiological properties of spinal MNs.

Lower mean body weights were observed in both groups of trained rats (*Bdnf*+/+ T and *Bdnf*+/− T) relative to their respective controls (7.6% and 14.6% decrease, respectively). The training regimen also impacted two fast hindlimb muscles (TA and MG) but did not affect the slow Sol muscle. The significant increases in the muscle-to-body weight ratio observed for the TA and MG muscles were accompanied by increased MYO/MB levels in the TA muscle, as well as elevated levels of IL-15 in the MG muscle, in both groups of trained rats (Figs. 2H, 3G). According to Underwood and Williams^41^ and Ordway and Garry^42^, elevated levels of MYO/MB in skeletal muscle occur in response to increased contractile activity, including progressive treadmill running^43^. In addition, Pedersen and Fabbraio^44^ and Lee and Jun^45^ reported that normal IL-15 expression levels in various muscles can be altered by endurance exercise, while Yang et al.^46^ showed an increase in MG IL-15 levels in obese rats after 8 weeks of treadmill training. We emphasize that the endurance regimen used in our study raised the levels of myokines in the TA and the MG muscles, but did not impact those in the Sol muscle in either the *Bdnf*+/+ T or *Bdnf*+/− T groups. This result suggests a stronger involvement of fast muscles in the increased motor activity that occurs during endurance training.

Significant post-training increases in BDNF and GDNF concentrations were observed in the TA muscle of trained wild-type rats as well as in the MG muscles of both groups of trained rats irrespective of genotype. Significant increases in Trk-B were also observed in both groups of trained rats (Figs. 2, 3F). Notably, the changes in the TA and MG muscles coincided with training-evoked increases in myokine levels (MYO/MB in the TA muscle and IL-15 in the MG muscle). These results are consistent with a report by Matthews et al.^12^, who showed that BDNF is produced by human skeletal muscles in response to increased contractions but not released into the peripheral circulation. Moreover, Wehrwein et al.^47^ showed that levels of GDNF increase in the hindlimb muscles of rats after four weeks of treadmill walk training or two weeks of forced running wheel training. Thus, it is reasonable to speculate that post-training increases in muscle BDNF and GDNF concentrations may be involved in training-induced changes in MN electrophysiological properties.

The endurance training regimen used in our study induced significant reductions in the input resistance of fast MNs in animals from the *Bdnf*+/+ T and *Bdnf*+/− T groups compared to their respective controls (*Bdnf*+/+ and *Bdnf*+/−). There were also trends toward increased rheobase values and the minimum and maximum currents required to generate rhythmic discharges in fast MNs after treadmill exercise (Table 1). It has been previously shown that MNs innervating fast muscle fibres have lower input resistance and higher rheobases than MNs innervating slow muscle fibres, leading them to be recruited later than slow MNs^32,48^. As a result, we conclude that our endurance training regimen resulted in reduced fast MN excitability in both wild-type and knockout rats. In turn, the fast MNs became even less likely to be recruited during motor activity, allowing the slow MNs to contribute even more to hindlimb muscle movements. To explain this unexpected result we suggest that fast MNs which were frequently recruited during the training could be transformed and were recognized as slow after the endurance training, while the least excitable fast MNs (with the lowest input resistance and the highest rheobase) were not recruited enough to provoke adaptations. This finding is partly in line with results reported by Beaumont and Gardiner^4^, who showed larger cell capacitances in fast MNs after endurance training, suggesting that MNs were larger. However, no effects on indices of excitability (rheobase, cell input resistance) were observed, and morphological studies in rats did not indicate changes in MN size after that the endurance training^49,50^.

The aforementioned results, together with the observed training-induced increases in muscle BDNF and GDNF, suggest that these NFs may be retrogradely and trans-synaptically transported to MNs. They may also contribute to reduced fast MN excitability by altering ion conductance and the expression of ion channels as hypothesized by Gardiner^29^. The possibility that BDNF regulates MN excitability has been previously demonstrated by Gonzales and Collins^32^, who observed changes in the excitability of fast MNs in rats after 5 days of continuous BDNF administration to the gastrocnemius muscle. However, there is a substantial discrepancy between the general direction of changes in the MN excitability observed in our experiments (a decrease) and the aforementioned result (an increase). This difference may be due to multiple factors. First, different BDNF induction methods were used (5 weeks of endurance training vs. 5 days of continuous exogenous application). Second, different concentrations of BDNF in the MG muscle were attained; the mean post-training concentration in our study was 0.9–1.0 ng/ml, whereas relatively high doses (8.0 and 16.6 mg/ml) of intramuscular BDNF were applied in the previous study. Third, repetitive activation of spinal MNs through feedback reflex loops from contracting muscles during running exercise may be an additional influence. Slow motor units are believed to be highly involved whereas the least excitable fast motor units are less active or remain inactive, but an additional activity time during running exercises would have negligible influence on slow motor units, as their average daily time is in rat muscles significantly longer than a daily activity time of two subtypes of fast motor units (5.3–8.4 h per day vs. 23–72 min for fast resistant and 0.5–3 min for fast fatigable units, respectively)^51^. This phenomenon may obscure the full range of changes that occur in various types of MNs.

In light of a report by Delezie et al.^52^, another possible explanation for our results is that BDNF is required for fibre-type specification in mouse skeletal muscle. Specifically, this previous report showed that BDNF overexpression promotes a fast muscle type gene program and elevates the number of muscle fibres with a glycolytic phenotype. This phenomenon could plausibly support our results, indicating that the observed decrease in fast MN excitability may be due to an increase in the number of glycolytic muscle fibres that these neurons innervate. However, it remains an open question whether such a transformation actually occurred in the muscles of any of the trained animals in our study.

Furthermore, we note that muscle-derived GDNF is a synaptotrophin responsible for maintaining synaptic connections^53^. Moreover, activity-related changes observed in the expression of skeletal muscle GDNF suggest that it may be involved in the modulation of neuromuscular junction architecture^47,54–57^. For example, Gyorkos et al.^58^ demonstrated that voluntary high-intensity running (with and without resistance) significantly increased GDNF content and end-plate area in the fast plantaris muscle of rats. Thus, the observed increases in GDNF in both fast muscles (the TA and MG) after moderate endurance training may facilitate communication between MNs and their muscle fibres.

We conclude that changes in the levels of specific skeletal muscle NFs evoked by moderately increased physical activity play an important role in spinal MNs’ plasticity, which is manifested by changes of certain electrophysiological properties of MNs, reflecting their excitability. In contrast, changes in the concentrations of circulating NFs do not induce any changes in MN properties.

## Methods

### Animals

This study was conducted on male Sprague–Dawley rats purchased from SAGE Labs (St. Louis, MO, USA). On average, the rats were 8 weeks old and weighed 298 ± 53 g upon arrival. Thirty animals were *Bdnf*-wild-type and 30 rats were heterozygous. *Bdnf* was knocked out using zinc finger nuclease technology (SD-BDNF). This technology uses artificial restriction enzymes generated by fusing a zinc finger DNA-binding domain to a DNA-cleavage domain. This design only targets unique genome sites and is unlikely to cause significant off-target effects, because the DNA binding motif specified by the zinc fingers directs the zinc-finger nuclease to a specific locus in the genome^59,60^. Therefore, other neurotrophins’ loci were not targeted in BDNF knockout rats. The animals were randomized to four groups, each consisting of 15 rats (*Bdnf*+/+, *Bdnf*+/−, *Bdnf*+/+ T and *Bdnf*+/− T). Due to diseases, four rats were excluded from the experiment, whereas ten animals that refused to participate in running exercises during the adaptation period were transferred to non-trained groups. As a result, the group sizes were as follows: *Bdnf*+/+: 19 rats, *Bdnf*+/−: 18 rats, *Bdnf*+/+ T: ten rats, and *Bdnf* + / − T: nine rats. Moreover, blood and muscle biomarkers could only be reliably measured in a proportion of samples, whereas stable electrophysiological recordings from individual MNs could not be obtained in some rats. The final group sampling taken for analysis was: 12 *Bdnf*+/+ rats, 11 *Bdnf*+/− rats, 9 *Bdnf*+/+ T rats, and 9 *Bdnf*+/− T rats.

The animals were housed in standard laboratory cages (2 rats of the same genotype per cage) with water and standard laboratory food available ad libitum. The room in which the animals were housed had controlled environmental conditions (a reverse 12 h:12 h light/dark cycle, 55 ± 1% humidity, and 22 ± 2 °C). The rats were acclimated to their environment for at least 7 days before starting the experimental procedures. Each rat was handled daily for approximately 15 min to reduce distress.

### Ethical approval

All experiments were approved by the Local Ethical Committee in Poznań (approval number 58/2018), and the research was conducted in strict compliance with the Polish Animal Protection Act and European Union regulations. This study was performed in accordance with ARRIVE guidelines.

### Endurance training protocol

Endurance training in the *Bdnf*+/+ T and *Bdnf*+/− T groups was performed on an electric treadmill for small rodents (Exer-6M, Columbus Instruments). The same training protocol, consisting of one week of adaptation and 5 weeks of regular running exercises, was performed for both groups. Each training session took place between 9 and 10 a.m. and was controlled by the same observer. During the adaptation period, all animals were acclimated to running on a motorized treadmill (10–45 min twice per day at a speed of 10–13 m·min^−1^). During the training period, animals ran continuously (45 min per day, 5 days per week for 5 weeks), with gradually increasing speed as follows: 15 m·min^−1^ in the first week (average daily distance: 675 m), 17.7 m·min^−1^ in the second week (average daily distance: 797 m), 19.3 m·min^−1^ in the third week (average daily distance: 869 m), 21.5 m·min^−1^ in the fourth week (average daily distance: 963 m), and 24 m·min^−1^ in the final week (average daily distance: 1080 m). According to Lalanza et al.^61^, the treadmill endurance running regimen used in our study is classified as moderate-intensity exercise.

### Electrophysiological experiments

Animals were deeply anaesthetized with intraperitoneal injections of sodium pentobarbital at an initial dose of 60 mg·kg^−1^ and supplemented with additional doses of 10 mg·kg^−1^·h^−1^ every hour. During surgery, the depth of anaesthesia was regulated based on a lack of pinna and withdrawal reflexes; during the recording sessions, the heart rate was monitored (300–360 beats·min^−1^). To paralyze the muscles and enable artificial ventilation during the recording sessions, pancuronium bromide (Pancuronium, Jelfa, Poland) was administered intravenously every 30 min (first dose: 0.4 mg·kg^−1^; supplementary doses: 0.2 mg·kg^−1^). From this point onwards, expired CO_2_ levels were monitored continuously (Capstar 100, CWE) and maintained at 3–4% by adjusting ventilation parameters (Small Animal Ventilator, SAR-830/AP, CWE). In order to minimize respiratory movements, a pneumothorax procedure was performed on the side of the recordings. The animals were euthanized with an overdose of sodium pentobarbital (180 mg·kg^−1^) at the end of the experiments.

The surgical procedure consisted of the following steps: (1) insertion of catheters into the right saphenous and femoral veins for blood sampling and drug administration, respectively; (2) endotracheal intubation for artificial ventilation; (3) left-sided preparation of the tibial nerve branch for further electrical stimulation; and (4) laminectomy over the L4–L6 spinal cord segments for insertion of recording electrodes. The animals were then placed in a metal frame and the vertebral column was stabilized by steel clamps. The dissected nerves and the exposed area of the spinal cord were then covered with paraffin oil. The animals’ core and oil temperatures were maintained within physiological limits (37° ± 1 °C) using an automatic heating system (Model 507222F, Harvard Apparatus). Subsequently, the dura was removed and small holes were made in the pia to insert glass microelectrodes into the spinal cord.

Bipolar silver ball electrodes connected to a square pulse stimulator (Model S88, GRASS Instrument Company) were used for antidromic stimulation of the tibial nerve (0.1-ms duration, amplitude up to 0.5 V, frequency of 3 Hz). Glass micropipettes with tips broken to 1.5–2.0 µm in diameter (10–15 Ohm) and filled with 2 M potassium citrate were used for intracellular recordings from single MNs located in lumbar spinal cord segments (L4–L5). Electrodes were inserted into the spinal grey matter using a step motor-driven manipulator with steps of 2–4 µm.

**Figure 5:**
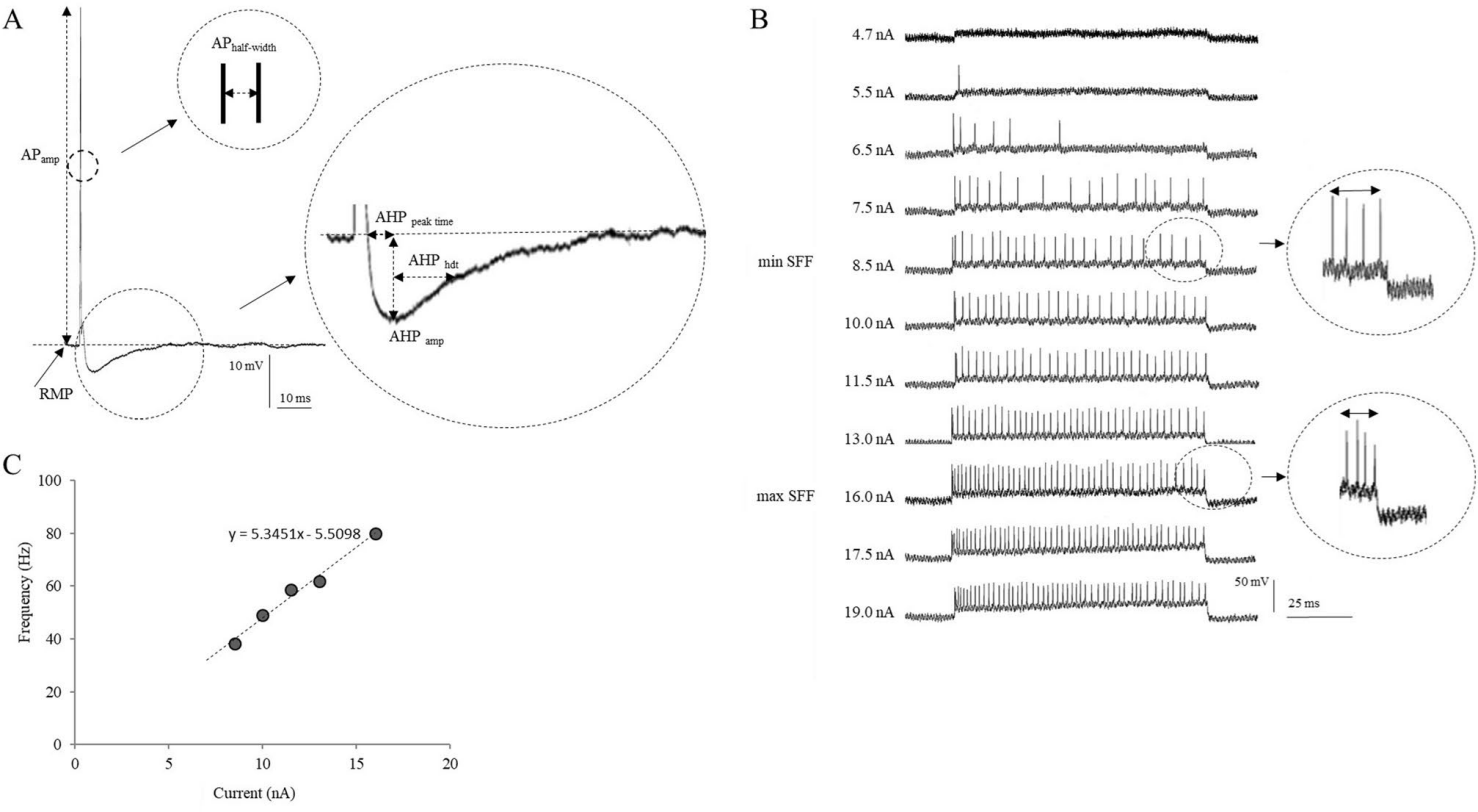
Examples of recordings obtained from a tibialis anterior MN from a *Bdnf* + / + rat. (**A**) Action potential parameters: RMP: resting membrane potential; AP_amp_: action potential amplitude; AP_half-width_: action potential duration measured at the level of half-amplitude; AHP_peak time_: time to afterhyperpolarization (AHP) peak; AHP_amp_: AHP amplitude; AHP_hdt_: AHP half-decay time. (**B**) Discharge patterns generated by a MN during a gradually increasing injection of intracellular current. Note the lack of rhythmic firing until intracellular stimulation at 8.5 nA (minimum steady-state firing [SSF] frequency: 38.2 Hz) and the maximum SSF frequency at 16 nA (80.0 Hz) without further increases at stronger intracellular stimulation levels. SSF frequencies were calculated from the last three inter-spike intervals (indicated with circles). (**C**) The frequency-current relationship (f/I) for the rhythmic firing in this MN, assessed based on the equation y = ax + b, where “a” determines the slope of the relationship.

MN recordings were acquired with an intracellular amplifier system (Axoclamp, model 2B, Axon Instruments) in bridge or discontinuous current clamp mode (current switch mode: 8 kHz) with capacitance maximally compensated and passed via a 16-bit analogue-to-digital converter (National Instruments, USB-6341) at a sampling rate of 10 kHz. A single MN was identified by antidromic stimulation of the tibial nerve, which innervates distal muscles of the hind limb (including the studied muscles: tibialis anterior, medial gastrocnemius and soleus). The antidromic nature of the recorded action potential was recognized on the basis of an all-or-none appearance and a stable, short-latency spike. Only stable recordings with resting membrane potentials of at least 50 mV and action potential amplitudes exceeding 55 mV with clear positive overshoot were considered. Twenty superimposed antidromic action potentials were automatically averaged by a custom laboratory computer program (BioLab) for further analysis. In the next step, 40 short pulses (100 ms) of hyperpolarization current (1 nA) were injected into each MN to measure input resistance (R_IN_). Intracellular depolarization current was later injected into individual MNs to induce an orthodromic action potential. From the averaged orthodromic spikes (obtained from 20 superimposed recordings), the following basic MN properties were calculated: resting membrane potential (RMP), action potential amplitude (AP_amp_), action potential duration measured at the level of half-amplitude (AP_half-width_), time to peak afterhyperpolarization (AHP_peak time_), AHP amplitude (AHP_amp_), and AHP half-decay time (AHP_hdt_) (Fig. 5A). The rheobase value (Rheo) was calculated as the minimum amplitude of depolarization current required to induce a single spike within 50 ms, and the voltage threshold (VT) was determined from the rheobase trace as the point at which the first derivative of the voltage reached 10 mV·ms^−1^^62^. Subsequently, 500-ms rectangular depolarizing currents of gradually increasing amplitudes (in steps from 0.1 to 2 nA) were applied intracellularly to individual MNs to induce rhythmic discharges. The minimum and maximum currents evoking SSF firing during the entire 500-ms recording window were identified and SSF frequencies were calculated from the averages of the last three inter-spike intervals (Fig. 5B). For each MN, a linear relationship between the SSF frequencies and the respective values of injected current was derived (f/I relationship) (Fig. 5C).

AHP_hdt_ (see Fig. 5A) is a MN property, which was experimentally proved to be a reliable tool to divide rat spinal MNs into fast and slow types. According to the method presented by Gardiner et al.^36^ the MNs with the AHPhdt shorter than 20 ms were classified as fast, while those with AHP_hdt_ equal or longer than 20 ms were classified as slow. Membrane and firing properties of MNs were further analyzed separately for each type.

### Biochemical analyses

At the beginning of each electrophysiological experiment, a fresh blood sample of 0.8 ml was taken from the saphenous vein and stored in test tubes. Then, blood was centrifuged for 5 min at 5000 rpm at 4 °C in order to separate and obtain serum. The serum samples were stored at −80 °C until analyses.

After the electrophysiological experiment, the right hindlimb TA, MG, and Sol muscles were dissected and any visible connective and fat tissues were removed. The prepared muscles were weighed and placed in cryogenic vials (NUNC/Thermo Fisher Scientific). The tissues were then frozen by immersion in liquid nitrogen and stored at −80 °C. The tendons from the TA and MG muscles were removed and cross-sections of the distal parts of these muscles were taken for homogenization. The Sol muscle was homogenized en bloc. Muscle samples were homogenized in phosphate-buffered saline (tissue to buffer ratio: 1:9) with EDTA-free Halt Protease Inhibitor Cocktail (100×; Thermo Fisher Scientific) to stop the protein lysis reaction. Homogenization was carried out using a dispersive homogenizer (VDI 12, VWR, Singapore) at 28,000–30,000 rpm in four 30-s cycles; cycles were separated by 1-min cooling breaks of ice water (4 °C). The homogenates were centrifuged (5000 rpm, 5 min, 4 °C) and supernatants were stored at −80 °C.

Concentrations of BDNF (sensitivity: 0.035 ng·ml^−1^; cat. number: SRB-T-81493), GDNF (sensitivity: 0.05 ng·ml^−1^; cat. number: SRB-T-85857), NGF (sensitivity: 0.276 ng·ml^−1^; cat. number: SRB-T-85674), NT-3 (sensitivity: 0.184 ng/ml; cat. number: SRB-T-85583), and NT-4 (sensitivity: 12.887 pg·ml^−1^; cat. number: SRB-T-85586) in both serum and muscle tissue were measured by ELISA according to the manufacturer’s recommendations (Sunredbio, China). ELISA was also used to measure concentrations of Trk-B (sensitivity: 12.337 pg/ml; cat. number: 201-11-0426), MYO/MB (sensitivity: 4.157 ng/ml; cat. number: SRB-T-84588), and IL-15 (sensitivity: 1.026 ng·L^−1^; cat. number: SRB-T-83418) levels in muscles. Absorbance was read at 450 nm using a multi-mode microplate reader (Synergy 2 SIAFRT, BioTek, Winooski, VT, USA).

### Statistical analysis

All statistical analyses were performed using Statistica 13 software (Statsoft, Cracow, Poland). Data were expressed as the mean ± standard deviation (SD) for MN parameters and concentrations of NFs and myokines. The data were analysed using a two-way analysis of variance (ANOVA) with genotype (*Bdnf*+/+ vs. *Bdnf*+/−) and training status (trained vs. untrained) as fixed factors. Tukey’s HSD post-hoc tests were performed to compare pairs of means. The levels of significance were set at p < 0.05, p < 0.01, and p < 0.001.

## Data Availability

The data that support the findings of this study are available from the corresponding author, WM, upon reasonable request.
